# Green Blends Based on Ionic Liquids with Improved Performance for Membrane Technology: Perspectives for Environmental Applications

**DOI:** 10.3390/ijms23147961

**Published:** 2022-07-19

**Authors:** Anca Filimon, Adina Maria Dobos, Oana Dumbrava, Florica Doroftei, Lavinia Lupa

**Affiliations:** 1 Polycondensation and Thermostable Polymers Department, “Petru Poni” Institute of Macromolecular Chemistry, Grigore Ghica Voda Alley 41A, 700487 Iasi, Romania; necula.adina@icmpp.ro (A.M.D.); dumbrava.oana@icmpp.ro (O.D.); 2 Physics of Polymers and Polymeric Materials Department, “Petru Poni” Institute of Macromolecular Chemistry, Grigore Ghica Voda Alley 41A, 700487 Iasi, Romania; florica.doroftei@icmpp.ro; 3 Faculty of Industrial Chemistry and Environmental Engineering, Politehnica University Timisoara, 6 Vasile Parvan Blv, 300223 Timisoara, Romania; lavinia.lupa@upt.ro

**Keywords:** quaternized polysulfone, ionic liquid, rheological properties, processing solution, membrane technology, diclofenac removal

## Abstract

Present research was directed towards the development of new high-performance and cost-effective polysulfone membranes (PSFQ) by introducing ionic liquids (ILs—Cyphos 101 IL and Aliquat 336) into their matrix. Variation of ILs was performed with the aim to find the one that brings new properties and improves the functionality and selectivity of PSFQ membranes in ultrafiltration processes. Based on the obtained results of the rheological study, we established the compatibility of compounds and optimal content of the used ILs, namely 3 wt% and 15 wt% Cyphos 101 IL and compositions varying between 3 and 15 wt % Aliquat 336. Results indicated that the ILs acted as plasticizers when they were added to the system, a helpful aspect in processing membranes used in water decontamination. The efficiency and performance of the membranes were evaluated by their use in the treatment of diclofenac (DCF)-containing waters. Membranes obtained from PSFQ/Aliquat 336 solution containing 15 wt% IL exhibited a 97% removal degree of DCF in the treatment process of 50 mL solution containing 3 mg/L DCF. The separation efficiency was kept constant for four filtration/cleaning cycles. The results indicated an improvement in membrane performance as the amount of IL in their structure increased, which confirms the potential for application in water treatment processes.

## 1. Introduction

Water represents the most crucial compound for life on Earth, and, therefore, clean and uncontaminated water is needed for life to exist. The population growth and the developments brought by the industrial revolution have led to a growing demand for water resources, and the deficit is now an indisputable result. Unfortunately, part of this water is polluted by industrial plants, mining, oil or gas exploration, fertilizer and pesticide residue used in agriculture, as well as by municipal wastewater and environmental and global changes [1,2]. Thus, overcoming problems created by the water deficit is a widespread public issue, and, for this reason, the identification of acceptable solutions for the recovery/reuse of wastewater is necessary.

In recent decades, pharmaceuticals and personal care products have been widely used around the world, and their continued release and corresponding metabolites have caused varying degrees of environmental pollution [3,4]. Different technologies for the elimination of hazardous chemicals from water [5,6], which include absorption, adsorption, distillation and membranes, have been developed. The selection of wastewater treatment systems depends on factors such as the method’s flexibility, process costs or the process’s environmental compatibility [7]. Nowadays, membrane-based separation technologies arose researchers’ interest, because membrane processes are governed by a pressure gradient [8], making them greener and more energy-efficient approaches and convenient operations compared to the traditional separation technologies mentioned above [9]. These separation technologies play important roles in industry and our daily lives, because of their functions in the concentration, fractionation and purification of liquid mixtures. However, most of the membranes are fabricated using toxic organic solvents or strong acids, which often generate toxic volatile organic compounds and produce a large amount of wastewater containing toxic solvents [10].

It is a huge challenge to reduce the amount of volatile organic compounds (VOCs) used in chemical and industrial processes. Therefore, the development of more efficient and environmentally friendly processes will be obligatory in the coming years. Research on chemical manufacturing has focused on the investigation of different approaches for diminishing the emission of VOCs, including solvent-free processes and the use of water and, more recently, ionic liquids (ILs) as the reaction media [11]. Among solvents, ILs have been rather sanguinely viewed as environmentally friendly or “green” solvents. To avoid these environmental problems, one direct strategy is replacing the common toxic solvents with greener solvents, such as ionic liquids (ILs). They have received extraordinary attention due to their excellent properties, such as strong polarity, high ionic conductivity, negligible vapor pressure (above the liquid surface), low volatility, thermal and chemical stability, designable structure and good ability to dissolve many inorganic, organic and polymeric materials [12,13,14,15]. Unlike the interesting and tunable properties of ionic liquids, their production cost at commercial scales is a major subject of investigation. As result, in industrial applications, combining ionic liquids with non-IL constituents to produce an optimum hybrid solution is an attractive strategy to reduce the costs [16]. These characteristics provide new opportunities to use membranes based on ionic liquids in membrane techniques that are more energy-efficient and environmentally friendly than the present commercial separation technologies.

In view of these aspects, it is generally recognized that a key role is played by the separation material since the correct material design, enhancing the separation efficiency and reducing the cost associated with material recycling and stability have a huge impact on the effectiveness of the separation technique. The global trends to protect the environment require the reconsideration of the possibility of using polymeric materials as supports/membranes, which could play an important role in addressing the environmental requirements, decontamination of aqueous systems and metal recovery. In this context, the scientific research in the membrane technology field [17,18,19] has highlighted the importance of polysulfones (PSF) due to their functional properties, being some of the best membrane materials. However, there are some deficiencies in their applicability in this field; the main drawback is given by the high hydrophobicity [20,21], which usually leads to an increase in the fouling probability of the membrane, generating an increase in the hydraulic resistance to water flow and, thus, decreased water permeability and economic efficiency, restricting its practical application in water treatment and, consequently, reducing the membrane lifetime [22,23]. For this reason, previous studies focused on the improvement of the PSFs’ properties, regarding the solubility characteristics [24,25,26], morphology and, consequently, the final surface properties (hydrophilicity, antimicrobial properties, higher permeability and better separation [20,27,28]). Thus, the functionalization of PSFs by chloromethylation [29] and quaternization [30] was used to guide, in a directed manner, the design of a practical membrane material with biomedical and environmental applications. Consequently, the development of advanced materials to improve current membrane technologies represents one of the foremost challenges. 

Thus, unlike previous studies, in this paper, we aim to provide designable membrane materials not only to enhance the process efficiency but also to deliver a cost-effective and environmentally friendly solution. For this purpose, polysulfone membrane materials based on ionic liquid were evaluated as a tunable platform for designing specific advanced materials for water treatment. The use of the structure–property relationship for these membrane materials enables the molecular control of the chemical functionalities by the introduction of structural features relevant for water treatment. In this context, in the present study, the ionic liquids, such as trihexyl (tetradecyl) phosphonium chloride (Cyphos 101 IL) and methyltrialkylammonium chloride (Aliquat 336), were selected due to their strong electrostatic characteristics and solvation forces, which lead to a more stable system. Based on their versatile properties, the distribution of the Cyphos 101 IL and Aliquat 336 ionic liquids through the polysulfonic chains is appropriate, suggesting a good fit in the polysulfonic membrane structure. The benefits of these ILs crossing within the PSF membrane materials are highlighted here by the solutions’ parameters, and the separation efficiency is also evaluated according to reference criteria. As an example of the application of these polysulfone blends based on ionic liquids, new membranes have been designed and developed, which were used in the treatment process for the membrane separation of waters containing diclofenac. Diclofenac is a non-steroidal anti-inflammatory drug (NSAID) and has been considered a persistent toxic pollutant in the environment. Even at very low concentrations, diclofenac can cause serious damage to the kidneys, liver and other human organs. On the other hand, residual diclofenac will also affect the development and growth of microorganisms [31,32]. For these reasons, this pollutant was selected to determine the efficiency of the studied polysulfone blends based on ionic liquids in water treatment processes through membrane filtration.

## 2. Results and Discussion

### 2.1. Rheological Response of Polysulfone Blends Based on Ionic Liquids 

The relaxation dynamics and structure–property relationships in polymer systems containing ionic liquids have been investigated using rheological measurements. Dynamic rheology is one of the most useful tools to assess the quality of ILs in the polymer solution, processed in the form of films, fibers, etc. Consequently, they help to verify the suitability of the obtained materials for the design and optimization of processing techniques before their production and utilization on a larger scale. Moreover, the rheological functions evaluated by the viscosity analysis as a function of shear rate and temperature represent the most important parameters in understanding the physical and structural changes, polymer−IL interactions, polymeric chain mobility and hydrodynamic volume of ILs dispersed in the polymer matrix [33,34]. For optimal performance, it is necessary to understand the rheological properties of IL—based systems, in terms of the thermodynamics and dynamics of the polymer chains in ILs, by analyzing the hydrodynamic dimension of the dispersed polymer, the plasticization efficiency and the overall properties of the final material. 

**Figure 1:**
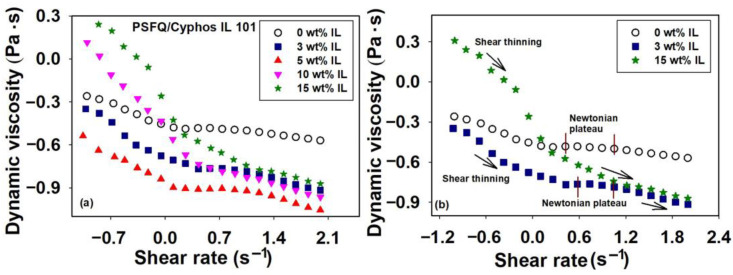
Rheological profile illustrated by double—logarithmic plots of dynamic viscosity (η) versus shear rate ($\dot{\gamma }$) for PSFQ/Cyphos IL 101 system at 25 °C and various mixing ratios, namely (**a**) Cyphos IL 101 compositions varying between 3 wt% and 15 wt% and (**b**) Cyphos IL 101 compositions of 3 wt% and 15 wt%, shown for a better visualization of the viscosity changes in the three regions.

**Figure 2:**
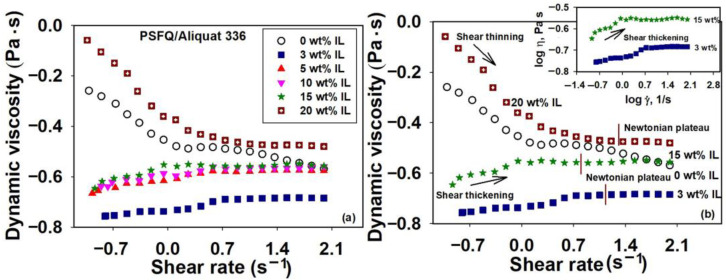
Rheological profile illustrated by double—logarithmic plots of dynamic viscosity (η) versus shear rate ($\dot{\gamma }$) for PSFQ/Aliquat 336 system at 25 °C and various mixing ratios, namely (**a**) Aliquat 336 compositions varying between 3 wt% and 20 wt% and (**b**) Aliquat 336 compositions of 3 wt%, 15 wt% and 20 wt%, for illustration of the viscosity changes in the two regions. Small image is inserted for better visualization.

In this context, the material’s response to the applied deformation, described by flow curves as a function of shear rate, for systems containing PSFQ, dissolved in N,N—dimethylformamide (DMF), and ionic liquids (trihexyl(tetradecyl)phosphonium chloride—Cyphos IL 101, and methyltrialkylammonium chloride—Aliquat 336) in various compositions (see Figure 1 and Figure 2), present complex behavior under the specific particularities of ILs. Thus, the examination of the flow curves in Figure 1 reveals that, in the case of PSFQ, the dynamic viscosity decreases in the range of low shear rates, followed by a region with constant values (Newtonian plateau) in the range of shear rates between 1.8 and 5.4 s^−1^; then, as the shear rate increases above this value, a slight decrease in dynamic viscosity occurs again. Similar rheological results were also observed for PSFQ—based solutions with low content of Cyphos IL 101 (i.e., 3 wt% and 5 wt% Cyphos IL 101). The same thinning behavior characterized the solutions with higher content of Cyphos IL 101 (i.e., 10 wt% and 15 wt% Cyphos IL 101), with the exception that in the range of low shear rates, the dynamic viscosity is higher than for PSFQ samples, and, starting from a certain shear rate of approximately 1.3 s^−1^, a continuous decrease in the dynamic viscosity occurs, without showing the Newtonian plateau, becoming smaller than that of PSFQ.

According to the results obtained, depending on the stress parameter value, the material structure undergoes reversible changes, reflected in the variation in viscosity highlighted in the rheological profile through three regions [35,36]. Thus, in the first region (Region I), when the viscosity decreases with increasing shear rate, a shear thinning regime (pseudoplastic behavior at low shear rates) occurs. The second region (Region II), characterized by a linear range, where the viscosity is independent of the shear rate, displays a Newtonian behavior (Newtonian plateau at intermediate shear rates), followed by another shear thinning regime (Region III) at high shear rates. Additionally, the literature [37,38] indicates that the non—Newtonian behavior manifested in Regions I and III can be explained by the tendency of the applied force to disturb the polymer chains from their equilibrium conformation, producing an elongation in the direction of shear. In other words, the thinning behavior is due to changes in the structure of the material that generate interactions between the polymer chains and dispersion medium. Thus, as the shear force increases and gradually becomes predominant, the polymer chains orient in the flow direction. In these conditions, shear flow could decrease the probability of interchain association at the expense of intrachain association, thus leading to lower viscosity [37].

Instead, for the system containing Aliquat 336, the rheological curves show only Regions I and II (Figure 2). Analyzing the obtained results, it was found that for the Aliquat 336 compositions varying between 3 wt% and 15 wt%, in the range of low shear rates, the dynamic viscosity continues to increase up to a shear rate of approximately 5.5 s^−1^, followed by a Newtonian plateau above this value. 

This non—Newtonian behavior, of a continuous increase in viscosity at low shear rates (Region I), is specific to shear thickening, which, according to the literature [39,40], is linked to two mechanisms, one associated with volume expansion and the second one with the formation of a network. The shear thickening behavior observed for the PSFQ/Aliquat 336 system can be attributed to the ionic packing and orientation in the bulk fluid during shearing action, in accordance with the statements of Freundlich and Roder [40]. The authors consider the shear thickening behavior a property that occurs under the action of shearing associations or granule packages, which are mobile in the liquid phase. The liquid inside the associations wets all the particles. By destroying this structure, new open spaces formed by granules arise, in which the liquid is aspirated; due to the local dehydration and direct friction between the particles, the whole system “stiffens”. After the stresses removal, the solution returns to its original state with an independent distribution of granules. In addition, this non-Newtonian behavior can be reversible or irreversible depending on the interaction between Aliquat 336 IL molecules and PSFQ chains in contact and also on the Aliquat 336 IL molecules’ alignment during shear [41,42].

During flow, the shear forces act in different ways: namely, they can form structures by alignment and association, or they can break intermolecular bonds that are not strong enough. The viscosity of the system will increase or/and decrease, respectively, depending on the direction in which the shear acts [43]. Rheological thickening in the PSFQ/Aliquat 336 IL system occurs when the frequency of collisions between the PSFQ groups and Aliquat 336 IL molecules increases and favors the formation of networks or cation−DMF−anion clusters and the squeezing out of DMF from clusters. As the volume of the liquid phase (DMF/Aliquat 336 IL) is large enough, the increase in the volume causes only an increase in viscosity without affecting the volume of the system. With high content of Aliquat 336 IL (i.e., 15 wt%; see Figure 2b for better visualization), the thickening behavior is manifested both by the increased volume and the dynamic viscosity. Moreover, it can be noted that, for these compositions, the dynamic viscosity increases with increasing IL content, but does not exceed the dynamic viscosity value of the PSFQ sample. Additionally, as the content of IL increases, the Newtonian regime moves slightly towards lower shear rates. 

On the other hand, it is observed that, as the Aliquat 336 IL content increases, exceeding 15 wt% IL (Figure 2), there is a change in flow behavior from shear thickening to a pseudoplastic behavior. This aspect is characterized by a continuous decrease in dynamic viscosity up to a certain shear rate, followed by a linear domain where the system has Newtonian behavior. The increase in the length of the alkyl chain resulted in increased viscosity and a non-Newtonian behavior, together with a decrease in the Brownian motion of the molecules. Compared to the PSFQ sample, at a composition of 20 wt% Aliquat 336 IL, the Newtonian regime appears at a higher shear rate. Instead, if we compare this system with the one that presents the highest composition of Cyphos IL 101 (i.e., 15 wt%; Figure 1b), it can be seen that the latter does not show the Newtonian flow on the studied shear field. A shear thinning behavior occurs across the whole range of shear rates studied for IL with longer alkyl chains, namely Cyphos IL 101 compared with Aliquat 336 IL, which was associated with the disruption in the interactive forces of ILs [44]. Therefore, the different flow behavior can be explained by considering the synergism between the structural properties of the PSFQ, namely the charge density and size of polycation, and also of the used ionic liquids, depending on the alkyl chain length and cation and anion nature. Moreover, molecular backbone flexibility, which, in turn, is expected to depend on their chain packing efficiency and specific interactions, leads to different flow behaviors. In addition to these parameters, interionic interactions generated by ILs, such as hydrogen bonding, also significantly affect the rheological properties, unlike conventional molecular solvents. 

The Ostwald–Waele model (Equation (1)) [45] was selected to fit the data obtained from the flow curves (Figure 1 and Figure 2).

(1) $$\sigma =k\cdot {\dot{\gamma }}^{n}$$

where the flow behavior (n) and consistency (k) indices were evaluated from the variation of the shear stress (σ) with shear rate ($\dot{\gamma }$).

**Figure 3:**
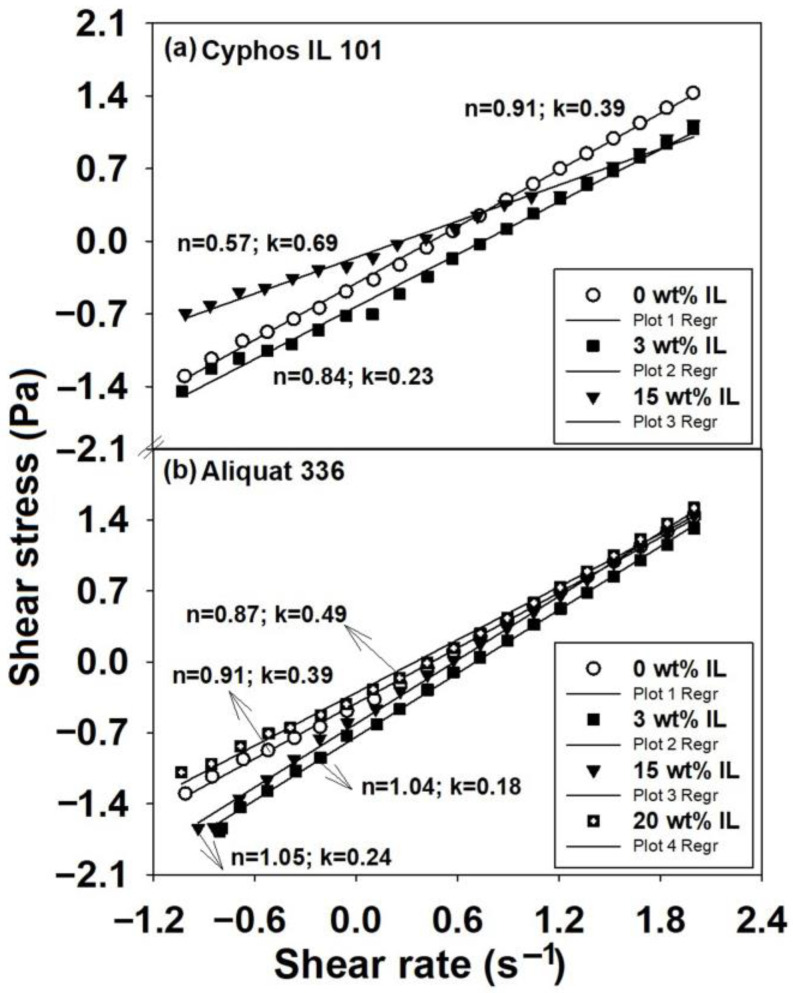
Ostwald–Waele model represented by double—logarithmic plots of shear stress versus shear rate for studied systems PSFQ/Cyphos IL 101 (**a**) and PSFQ/Aliquat 336 (**b**) at various mixing ratios and 25 °C.

The different trends of the Ostwald–Waele plots showed the complex behavior of the studied solutions. In this sense, the flow behavior is reflected in the values obtained for power law exponents, namely the n and k indices, according to Figure 3. 

Thus, the power law exponents displayed a variation as a function of the increase in IL content and also of its nature. Therefore, for PSFQ/Cyphos IL 101 systems at different compositions, the power law indices, n, take values lower than unity, revealing a shear thinning behavior (pseudoplastic). Flow behavior indices are close to unity for PSFQ samples and 3 wt% Cyphos IL 101, respectively, highlighting the Newtonian behavior, in accordance with the rheological behavior illustrated in Figure 1. Any increase in the composition of Cyphos IL 101, exceeding 5 wt% content, is shown by the decrease in the values of the flow behavior index. This decrease in the n reveals a shear thinning behavior that is much more pronounced over the shear rate range without a Newtonian regime, as compared to the PSFQ and PSFQ solutions with low content of Cyphos IL 101. Instead, according to Figure 2, the PSFQ/Aliquat IL system exhibits two distinguished types of behavior, characterized by different slopes (see Figure 3b). Therefore, in the first stage, for IL content varying between 3 wt% and 15 wt% Aliquat 336, the n power law index takes values higher than unity, revealing a shear thickening behavior. Subsequently, as we increase the Aliquat 336 IL content, exceeding 15 wt%, the n value decreases below unity, indicating a shear thinning behavior, according to Figure 2.

On the other hand, for the Cyphos IL 101—based systems, the consistency index with the lowest value is obtained for 3 wt% IL, which indicates that this represents the composition for which IL acts as a plasticizer. As the IL content increases, the consistency index increases, exceeding the value for the PSFQ sample, as follows: k_15 wt%_ > k_0 wt%_ > k_3 wt%_. Similar to the system consisting of Cyphos IL 101, in the case of the PSFQ/Aliquat 336 system, the consistency index has equally low values, which indicates that the IL acts as a plasticizer for small compositions. As the Aliquat 336 IL content increases, k slightly increases, and at a composition of 20 wt% IL, corresponding to the pseudoplastic behavior, the consistency index exceeds the corresponding PSFQ value, as shown by the variation: k_20 wt%_ > k_0 wt%_ > k_15 wt%_ > k_3 wt%_. In this context, one can conclude that the PSFQ—based IL systems possess properties of pseudoplastic materials, characterized by reduced entanglement density and an enhanced number of oriented segments, as a result of shear rates increasing. The higher orientation of the polymer chains is the major cause of the non-Newtonian behavior [37,46]. The existence of a pronounced shear thinning behavior is due to the creation of agglomerated structures in concentrated solutions [47]. Therefore, the obtained rheological profiles as a function of IL content, for both systems, can help to analyze the plasticization efficiency and the final properties of the studied systems, suggesting that these act as plasticizers. 

It is known that the presence of a plasticizer in a polymeric system causes a decrease in the glass transition temperature (T_g_) and an increase in system flexibility [48]. Moreover, the specific interactions developed by hydrogen bonding or/and the formation of structures by alignment and association increase T_g_ [49]. According to the above statements, the specific behavior evidenced in the rheological measurements can be confirmed by analysis of the glass transition temperature values obtained by the differential scanning calorimetry studies (DSC). Thus, due to the structure containing an aromatic core and pendant alkyl groups, the ILs can be used as plasticizers as a result of their excellent advantages, such as high thermal stability and their adjustable polarity and acidity. They are environmentally friendly, present small evaporation losses and also good compatibility with polymers, so they broaden the processing field by lowering T_g_ and enhancing the elasticity range [50,51]. Instead, the polysulfones are a special class of engineering polymers with high mechanical and thermal strength [52]. The thermal stability of the whole polymer is the result of the phenyl and sulfonyl groups, which are thermally stable [53]. Moreover, the sulfonyl groups are related to the aromatic system through resonance, so the inter-polymer bonds gain strength from the resonance energy. Therefore, higher degrees of thermal or radiation energy can be adsorbed by the molecular structure without further reaction or decomposition [53]. On the other hand, the thermal stability of the membranes containing ionic liquid is lower than that of the pure polymer membrane because the ionic liquid tends to decompose before the polymer matrix [54]. In accordance with the results mentioned, the T_g_ values of the PSFQ/Cyphos IL 101 system are lower (T_g_ values varying between 199.54 and 195.70 °C) than those of PSFQ (T_g_ = 201.53 °C), which indicates that the system has improved flexibility due to the addition of IL. Thus, the increasing ionic liquid content reduces the thermal stability. Instead, the addition of Aliquat 336 IL in various mixing ratios leads to a slight increase in T_g_ (T_g_ values varying between 202.25 and 205.10 °C) in comparison with PSFQ and the PSFQ/Cyphos IL 101 system. This aspect could be caused by the formation of a network/interaction developed as a result of the phenomenon of volume expansion, accompanied by the stiffness of the polymer chain due to the IL nature (methyltrioctylammonium chloride), as confirmed by the rheological study. Therefore, the results allow us to conclude that these compositions are optimum and lead to specific properties that make them suitable for future investigations concerning the development of performant membranes used in environmental applications.

### 2.2. Tuning of Microstructure in Polysulfone Blends Based on Ionic Liquids by Location of the Compatibility Limit 

**Figure 4:**
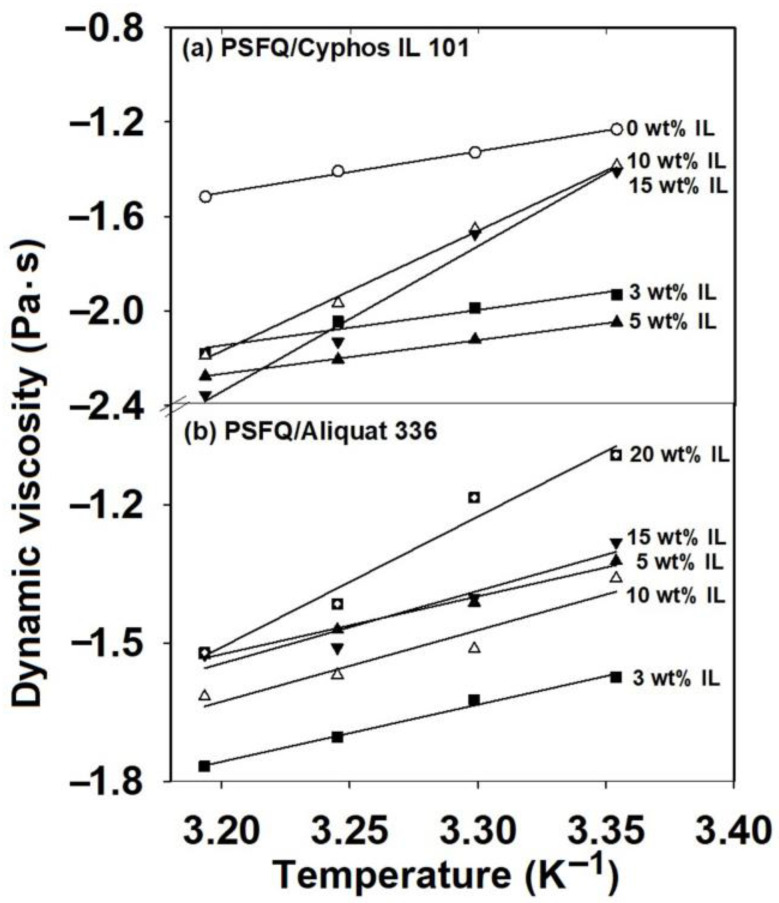
Dependence of the dynamic viscosity on temperature for (**a**) PSFQ/Cyphos IL 101 and (**b**)PSFQ/Aliquat 336 systems at various mixing ratios.

**Figure 5:**
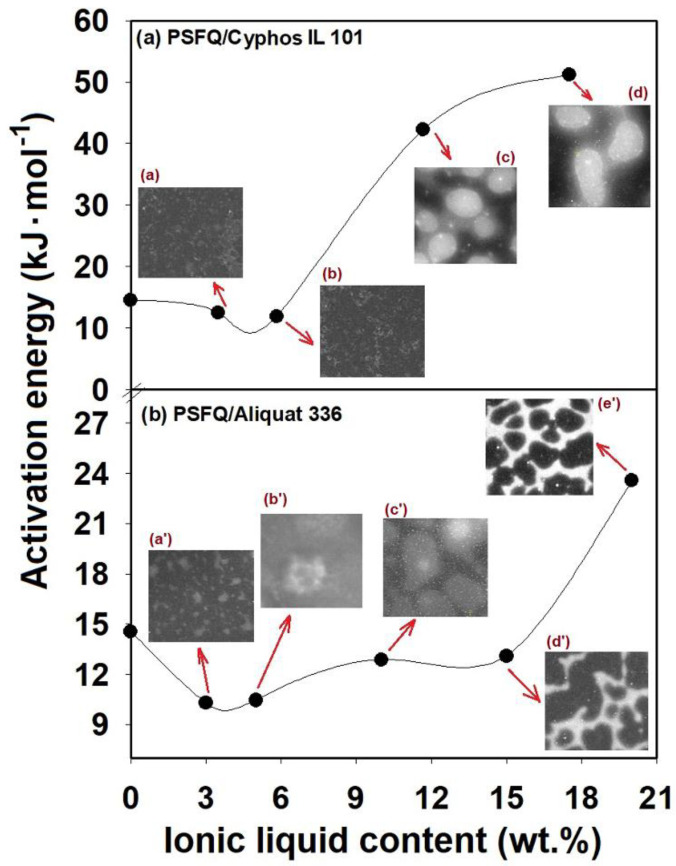
Activation energy as a function of IL composition for (**a**) PSFQ/Cyphos IL 101 and (**b**) PSFQ/Aliquat 336 systems. Small images represent STEM images recorded for different IL compositions in studied systems: (a) 3 wt%, (b) 5 wt%, (c) 10 wt% and (d) 15 wt% for PSFQ/Cyphos IL 101 system, and (a’) 3 wt%, (b’) 5 wt%, (c’) 10 wt%, (d’) 15 wt% and (e’) 20 wt% for PSFQ/Aliquat 336 system.

It can be stated that regardless of the nature of the IL (trihexyl(tetradecyl)phosphonium chloride (Cyphos IL 101) or methyltrioctylammonium chloride (Aliquat 336)), the partial replacement (3−20 wt%) of the conventional solvents with ILs showed a change in rheological behavior, from shear thinning (pseudoplastic) or shear thickening behavior to almost Newtonian behavior, described by the flow curves and reflected also in the values of the power law exponents. This indicates that ILs act as plasticizers when they are added to the system in low content and is indicative of the improvement in solution processing by their addition. Additionally, it can be assumed that this behavior represents the consequence of the reduced entanglement density and enhanced number of oriented segments, as result of shear rates increasing. All these results can be explained based on the modification of the specific interactions of the systems generated by the IL components. Therefore, the effect of the combined action of different thermodynamic interactions from the system, as well as the conformational changes in PSFQ in the presence of each type of IL in the blends, modifies the flow activation energy, E_a_ (Figure 4 and Figure 5). This parameter was evaluated with the Arrhenius equation [55] (Equation (2)) and derived from the dependence of dynamic viscosity versus temperature (Figure 4).

(2) $$\ln\eta ={\ln\eta }_{0}+E_{a}/\mathrm{RT}$$

where $\eta_{o}\;$~ e^Δ^^S/R^ represents a pre-exponential constant, ΔS is the flow activation entropy, R is the universal gas constant, and T is the absolute temperature. 

**Figure 6:**
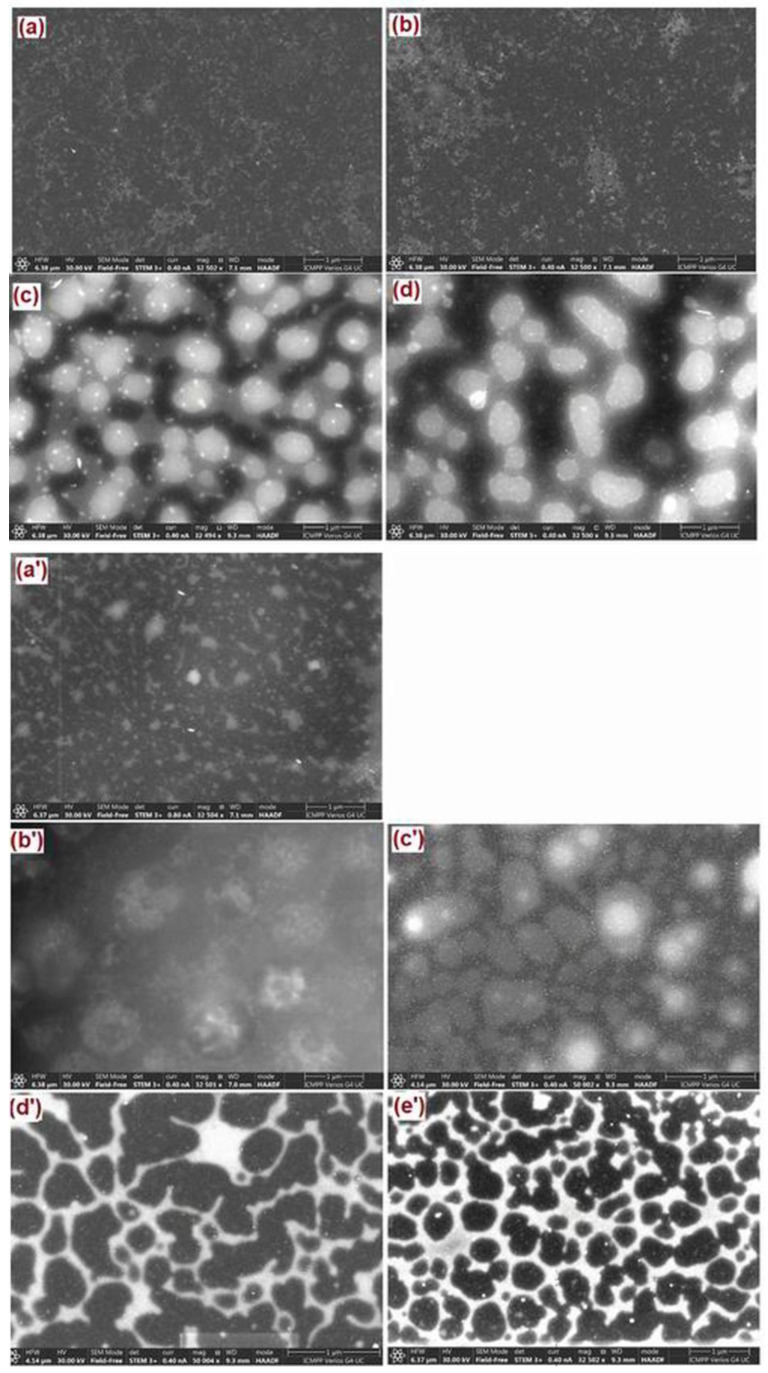
STEM images of the studied systems at different mixing ratios: (**a**) 3 wt%, (**b**) 5 wt%, (**c**) 10 wt% and (**d**) 15 wt% for PSFQ/Cyphos IL 101 system, and (**a’**) 3 wt%, (**b’**) 5 wt%, (**c’**) 10 wt%, (**d’**) 15 wt% and (**e’**) 20 wt% for PSFQ/Aliquat 336 system, recorded for 1 μm scan area and magnification of 32,500×.

The origin of the high complexity of these systems lies in the combination of the properties generated by the long alkyl chain molecules with those derived from the charge interactions that can induce localized aggregation [56] (Figure 5). As is known from the literature [57,58], the location of a system’s heterogeneous region is dependent on the combination of its components and their characteristics, which reflects a limitation of compatibility and the well-established compatibility domain. This aspect allows the evaluation of the miscibility gap and, implicitly, allows us to gain information on the thermodynamic stability. In this context, as a consequence of the used ILs’ impact on the PSFQ/DMF system, different thermodynamic stabilities can be expected. This is why, in this study, the influences of the selected ILs, in various compositions, were investigated comparatively, in two different systems, using scanning electron microscopy images (STEM) (see small images in Figure 5 and Figure 6).

As a function of composition, the occurring mechanisms are dependent on the entry point into the miscibility gap [59]. Thus, it could be seen that, added in small quantities, regardless of its nature—Cyphos or Aliquat—the ILs in the PSFQ solution led to a bi-continuous, uniform and regular structure (Figure 6a,b,a’). As the IL composition increases (see Figure 6c,d,b’,c’), the system directly enters the metastable region, and binodal decomposition occurs, which induces the formation of a closed-cellular, an open-cellular or a nodular morphology.

The stable systems were obtained due to the solvation forces of the ILs, but, according to the rheological profiles, some systems displayed a shear thickening characteristic (Figure 2), a phenomenon that was linked with the destruction of the solvated structure at the IL’s surface in the intermediate shear rate range. This fact resulted in the aggregation and development of interconnected networks of ILs (Figure 6b’,c’). In contrast, spinodal decomposition occurs when the system contains a large amount of IL (Figure 6d’,e’), moving from a stable state and the entering into the miscibility gap directly through the critical point into the unstable region [59,60]. Therefore, the structuring of the ILs, resulting from the strong electrostatic characteristics and the network between the ions, which drive the solvophobic segregation of the alkyl chains, was the controlling parameter for the shear thinning behavior, which shifted toward the higher shear rates due to the reduction in aggregates [61,62]. 

Consequently, investigations based on the solution analysis have demonstrated that the addition of ILs to the PSFQ solution changes the solvent quality, which leads to variations in the rheological behavior of the polymer solution, as well as changes in the thermodynamic nature of their phase separation. Therefore, we can conclude that these systems represent the optimum compositions where the distribution of ILs through the polysulfonic chains is appropriate, and they also possess specific properties, suggesting a good fit in the polysulfonic membrane structure, as predicted. Additionally, the choice of the IL/organic solvent system has a strong impact on the properties of the polymer solution, which consequently changes the diffusional processes during membrane formation [63].

### 2.3. Membrane Efficiency in the Treatment Process of Water Containing Diclofenac

As was observed from the study in the solution of polysulfone systems based on ionic liquids, their addition modifies the membranes’ characteristics (see Table 1). Thus, viscosity variations through compositional changes have a high impact on the diffusion rate [64]. Therefore, according to our results, to obtain a good ultrafiltration membrane for water treatment, it is necessary to ensure high pore density and, consequently, high permeability, which would highlight their suitability as selective barriers that allow the free transport of water vapor through the pores. In this context, the membranes obtained starting from polysulfone blends based on ionic liquids were tested in the filtration process of waters with diclofenac content.

According to data listed in Table 1, the pure water permeability, water content percentage and general porosity increase with the increase in the IL content in the membrane. Higher values of the studied parameters were obtained when using the PSFQ/Aliquat 336 system compared to the PSFQ/Cyphos IL 101 system.

The best measure of a membrane’s ability to separate wastewater molecules (*i*) is the permeability ratio α*_i_*, called membrane selectivity, which can be written in terms of the apparent separation coefficient, R [27,28,67] (Equations (3) and (4)):

(3) $$\alpha_{i}=\frac{C_{\mathrm{ip}}}{C_{\mathrm{if}}}$$

(4) $$R\left(\%\right)=\left(1-{\;\alpha }_{i}\right)\cdot 100$$

where C_if_ and C_ip_ represent the concentration of DCF in the feed solution, respectively, in the permeate (mg·L^−1^).

**Figure 7:**
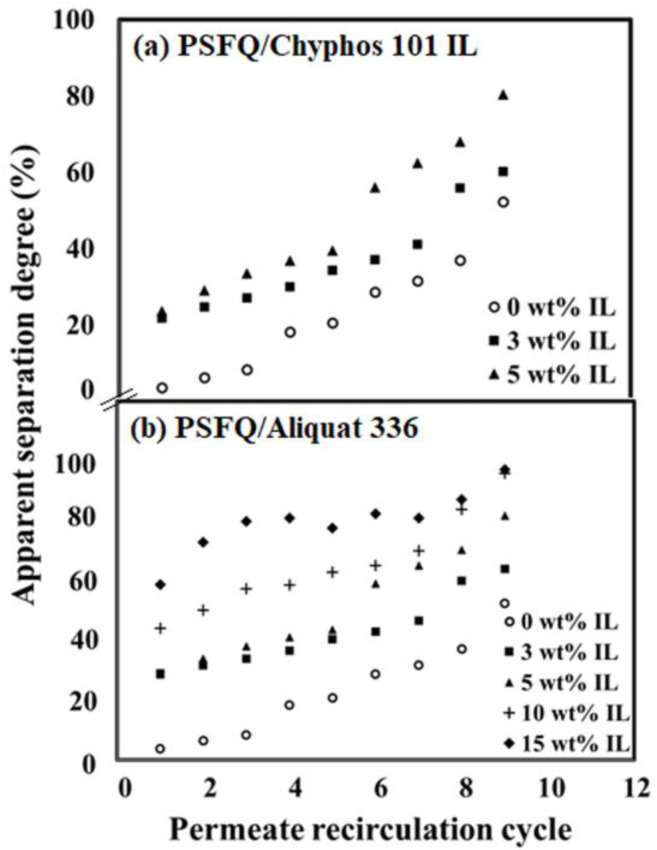
Apparent separation coefficient developed by the studied membranes, obtained from PSFQ/Chyphos 101 IL (**a**) and PSFQ/Aliquat 336 (**b**) blends, in the treatment process of waters containing 3 mg·L^−1^ DCF.

The experimental results regarding the dependence of the apparent separation coefficient, R, developed by the studied membranes in the treatment process of waters with 3 mg·L^−1^ diclofenac content, as a function of the permeate recirculation cycle are presented in Figure 7. It could be observed that with the increase in the permeate recirculation cycle over the studied membranes, the apparent separation coefficient increases for all the studied membranes. As the IL composition increases, a higher rejection degree is obtained. In the case of the membranes obtained from the PSFQ/Aliquat 336 system, this is obtained after nine cycles of permeate circulation over membranes of 97% when the content of IL in the system is 15 wt%. Comparing the studied systems, it could be observed that for a content of 3 wt% IL, we could obtain an apparent separation coefficient of 60.25% for PSFQ/Cyphos IL 101 and 63.9% for PSFQ/Aliquat 336, respectively. Increasing the IL content from 3 to 5 wt%, the apparent separation coefficient increases to 80.45% for PSFQ/Cyphos IL 101 and to 81.73% for PSFQ/Aliquat 336, respectively.

The membranes obtained from the polysulfone blends based on ammonium ionic liquids present slightly higher efficiency compared with those developed by the membranes obtained from the polysulfone blends based on phosphonium ionic liquids. The higher affinity of diclofenac for ammonium ionic liquids could be attributed to the greater electrostatic or intermolecular interactions between the diclofenac salt and ammonium cation compared with the phosphonium cation [68,69].

Following the obtained results, and due to the improvement efficiency of the membranes obtained from the PSFQ/Aliquat 336 solutions, they were used in the treatment process of water with higher diclofenac content (5 and 10 mg L^−1^). The amount of DCF accumulated in the membrane was determined using the following equation:

(5) $$Q_{\mathrm{ac}}=\frac{\left(C_{\mathrm{if}}-C_{\mathrm{ip}}\right)\cdot V}{A}$$

**Figure 8:**
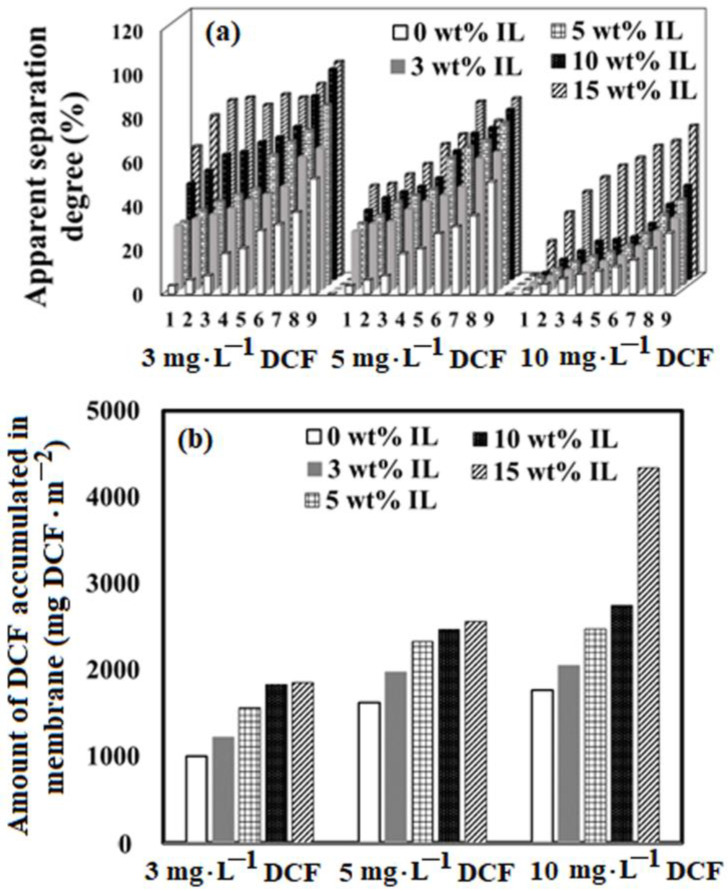
The rejection degree of DCF developed by the studied membranes (**a**) and the amount of DCF accumulated on the membranes at the end of the treatment cycles (**b**) for PSFQ/Aliquat 336 system.

The efficiency developed by the studied membranes for all three diclofenac solutions is presented in Figure 8.

It is observed that the behavior of the studied membranes is constant even if they are used with a higher initial concentration of DCF in the aqueous solutions; their efficiency increases with the increase in the permeate recirculation cycles and with the increase in the IL content. Thus, the apparent separation coefficient decreases with the increase in the initial concentration of DCF, but the amount of accumulated DCF on the membrane shows an increase. The apparent separation coefficient increases with the permeate recirculation cycle for the studied membranes and for all the treated waters, regardless of the DCF concentration. Even if the degree of rejection decreases with the increasing initial concentration of DCF in aqueous solutions, the amount of DCF accumulated on the membranes increases. These findings suggest that both the nature of the functional group of the used ionic liquid and the membranes’ structure through their porosity contribute to the effectiveness and performance of the membranes in the water treatment process.

**Figure 9:**
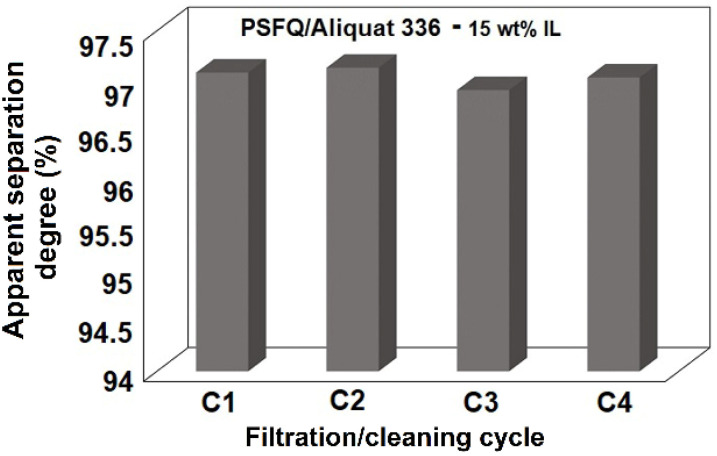
Membrane efficiency developed in 4 filtration/cleaning cycles for treatment process of 50 mL solution containing 3 mg·L^−1^ DCF.

The membrane obtained from the PSFQ/Aliquat 336 system, containing 15 wt% IL, was cleaned by backwash with distilled water and used in other filtration processes for 50 mL waters containing 3 mg·L^−1^ DCF. Four cycles of filtration/cleaning were performed and it could be observed that the membrane efficiency remained constant for all cycles (Figure 9).

It can be stated that the efficiency of the membranes is significantly improved by the presence of ionic liquids in the membrane structure, this improvement being much more obvious with the increase in the ILs’ content.

## 3. Materials and Methods

### 3.1. Materials

Commercial aromatic polysulfone (PSF, UDEL-3500, Union Carbide Company, Texas), with number-average molecular weight M_n_ = 39,000 g·mol^−1^, was used in the synthesis of functionalized polysulfones [29,30]. Cationic polysulfone containing quaternary ammonium side groups (PSFQ, M_n_ = 28,000 g·mol^−1^, ionic chlorine content 5.44%; Table 2) was synthesized by reacting chloromethylated polysulfone (CMPSF) with a tertiary amine, namely N,N—dimethylbutylamine, according to the detailed procedure presented in previous studies [29,30,70].

Trihexyl(tetradecyl)phosphonium chloride (Cyphos IL 101, purity > 97%) and methyltrialkylammonium chloride (Aliquat 336, mixture of tri-alkyl methyl ammonium chloride salts, with 88.2–90.6% quaternary ammonium content) are commercially available ionic liquids (purchased from Sigma-Aldrich, Darmstadt, Germany), used in the present study (Table 2). All chemicals, with the exception of PSF and synthetized polysulfones, were used as received, without any further purification. Chemical structures have been generated by ChemDraw Ultra 12.0 (CambridgeSoft Corporation, CambridgePrak Drive, Cambridge, MA, USA).

### 3.2. Membrane Preparation 

To obtain polymer inclusion membranes, initially, the homogeneous PSFQ solutions with various concentrations were prepared by dissolving them in N,N—dimethylformamide and, subsequently, by adjusting the volume ratios of the prepared PSFQ solutions and ionic liquids (i.e., 3–20 wt% Aliquat 336 and 3–15 wt% Cyphos IL 101), and PSFQ/IL blends with various compositions were obtained. All membranes used in this study, with thickness around 40 μm, were achieved by the solution-casting method. The above-prepared solutions were cast on a smooth Teflon substrate using an Automatic Film Applicator Standard (Perforated Heated Vacuum Bed-230 VAC, TQC Sheen, Netherlands) equipped with digital micrometer, and after casting, they were solidified initially by slow drying in a saturated atmosphere with the used solvent. Finally, to control the solvent evaporation rate, the deposited polymer blends were gradually oven-dried at different temperatures, and then, after 2 days of exposure in a controlled vacuum oven at 50 °C, porous membrane formation was induced. Additionally, the porous membrane preparation was considered definitive when the polymeric films were detached from the support.

### 3.3. Characterization and Testing

#### 3.3.1. Rheological Investigations

Most often, the polymer processing starts from the solution phase; thus, the rheological properties of the PSFQ/IL systems were evaluated and controlled under the influence of the composition, in correlation with the structural peculiarities and properties of the PSFQ and used ILs (Aliquat 336 and Cyphos IL 101), respectively. The rheological characteristics of PSFQ/IL blends in DMF were investigated with a CS50 Bohlin rheometer, manufactured by Malvern Instruments, equipped with cone-plate geometry (cone angle of 4° and diameter of 40 mm). Shear viscosities were registered over the 0.1–100 s^−1^ shear rate domain, at a temperature of 25 °C. Rheological tests were obtained with an accuracy of ±5%, for different measurements.

#### 3.3.2. Differential Scanning Calorimetry

The glass transition temperatures of the PSFQ and PSFQ/IL blends obtained in various mixing ratios were measured with a STA 449 F1 Jupiter instrument (Netzsch, Selb, Germany). The detailed procedure of the experiment was presented in a previous study [71].

#### 3.3.3. Scanning Electron Microscopy

The morphology of the samples was investigated with a scanning electron microscope of type Verios G4 UC (Thermo Fisher Scientific, Brno-Černovice, Cehia) working in STEM mode at 30 kV, with a STEM 3 + detector (bright-field mode). The samples were prepared on carbon-coated copper grids with 300-mesh size. Microdroplets of the PSFQ/ILs dispersed in DMF (0.1%) were placed on the grids, and then the solvent was removed under vacuum.

#### 3.3.4. Ultrafiltration Tests

The prepared membranes were thoroughly washed and cut to the desired size and loaded into an ultrafiltration test cell. The surface area of the membranes was 0.785 cm^2^ and they were initially pressurized to 3 Ba. The pressurized membranes were used in subsequent ultrafiltration experiments. The water content of the membranes was obtained after soaking the membranes in water for 24 h. They were weighed after being wiped with filter paper. The wet membranes were placed in an oven at 75 °C for 48 h, cooled to room temperature in a desiccator and weighed again. Water content percentage retained by the membrane, WC, was calculated based on the wet and dry membrane weight data, w_1_ and w_2_, respectively.

Subsequently, the studied membranes were used in the treatment of 50 mL of aqueous solutions containing 3 mg·L^−1^ DCF. Crossflow filtration was used, the feed solution being passed over the studied membrane using a vacuum of 20 mBar, recirculating the permeate through the membranes for 9 cycles. The studies were performed at the natural pH of the solutions (pH ≈ 6, T = 23 °C). The concentration of DCF in the feed solution and in permeate was determined by UV–VIS spectrophotometry at the wavelength λ = 275 nm, using a Varian Cary 50 spectrophotometer (Agilent, Santa Clara, CA, USA).

## 4. Conclusions

This work describes a method for the development of membranes using ionic liquids because they are cost-effective and environmentally friendly, properties that make them good alternatives for the conventional solvents, and they also enhance the membrane elasticity. In this context, the ability to obtain the desired membranes with well–established surface properties and separation efficiency based on rheological parameters was highlighted. Thus, it was demonstrated that the inclusion of different amounts of ionic liquids in quaternized polysulfone solutions changes the properties of these polymers. For this purpose, a detailed investigation was performed on the solution properties of PSFQ systems based on ionic liquids, namely trihexyl(tetradecyl)phosphonium chloride (Cyphos IL 101) and methyltrialkylammonium chloride (Aliquat 336), by a rheological study. Rheological data are imperative to obtain proper control and to establish the optimal compositions and quality of ionic liquids for future possible applications.

According to the obtained rheological profiles for both studied PSFQ–based IL systems, we concluded that, due to the structural characteristics of the used ILs and also to their good compatibility with PSFQ, they possess the properties of pseudoplastic materials. Consequently, investigations based on the solution analysis demonstrated that the addition of ILs to the PSFQ solution changed the solvent quality, which led to variations in the rheological behavior of the polymer solution, as well as changes in the thermodynamic nature of their phase separation. Enhancement of the chemical functionalities by the introduction of ILs, plasticization efficiency and adjustment of the system’s composition led to the achievement of the desired rheological optimum (elasticity/viscosity) and, implicitly, to the optimization of the processing parameters/techniques of the desired membranes.

As an initial attempt to evaluate the separation efficiency, the membranes obtained from PSFQ blends based on ILs were applied in the treatment process through the membrane filtration of waters with diclofenac content. The separation efficiency of the studied membranes increased with an increase in the ionic liquid amount in the membrane structure. However, the membrane obtained from the PSFQ/Aliquat 336 system, containing 15 wt% IL, exhibited a 97% removal degree of DCF in the treatment process of a 50 mL solution containing 3 mg·L^−1^ DCF. The separation efficiency remained constant for four filtration/cleaning cycles. These findings are in agreement with the rheological data obtained as a result of the slightly higher impact of ammonium ionic liquids (Aliquat 336) on the PSFQ matrix compared to that of phosphonium ionic liquids.

Therefore, the results allow us to conclude that these compositions are optimal and lead to specific properties that make them suitable for future investigations concerning the development of performant membranes based on ionic liquids, used in environmental applications such as water treatment through ultrafiltration processes.

## Figures and Tables

**Table 1:** The parameters of the studied membranes *.

System	Mixing Ratio (wt%/wt%)	J_w_ (L·m^−2^·h^−1^)	w_1_ (g)	w_2_ (g)	WC (%)	ɛ (%)
Equations		$\frac{V}{\Delta t\cdot A}$			$\frac{w_{1}-w_{2}}{w_{1}}\cdot 100$	$\frac{w_{1}-w_{2}}{A\cdot l\cdot \rho }$
PSFQ/ Cyphos IL 101	0	1529	0.0232	0.0308	24.7	2.43
	3	1592	0.0228	0.0319	28.5	2.90
	5	1698	0.0245	0.0615	60.2	11.8
PSFQ/ Aliquat 336	0	1529	0.0232	0.0308	24.7	2.43
	3	1663	0.0288	0.0492	41.5	6.51
	5	1779	0.0242	0.0636	61.9	12.6
	10	1819	0.0251	0.0689	63.6	13.9
	15	1930	0.0254	0.0760	66.5	16.2

* Membrane parameters including the pure water permeability, J_w_ [65] (knowing the amount of permeate collected (V, L), collection time (Δt, h), membrane surface area (A, m^2^)), water content of the membranes, WC [65], based on the wet and dry membrane weight data, w_1_ and w_2_, respectively, and general porosity, ɛ [66] (knowing *ρ* the density of water (0.998 g·cm^−3^), and l, the thickness of the membrane (μm)).

**Table 2:** Chemical structures of the monomer unit of polysulfone with quaternary ammonium groups (PSFQ) and used ionic liquids (trihexyl(tetradecyl)phosphonium chloride—Cyphos IL 101 and methyltrialkylammonium chloride—Aliquat 336).

**Polymer**	
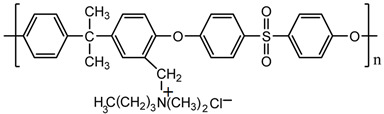	PSFQ
**Ionic Liquids**	
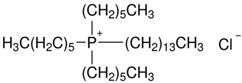	Cyphos IL 101
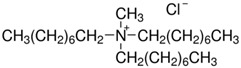	Aliquat 336

## Data Availability

Data supporting the findings of this study are contained within the article and are available from the corresponding author upon request.
